# Globally famous, locally recognized: Cross‐cultural validation of the use of Famous Faces Test in Croatia

**DOI:** 10.1111/jnp.70047

**Published:** 2026-04-17

**Authors:** Maja Kolanović, Mirta Stantić

**Affiliations:** ^1^ Department of Psychology University of Zagreb Zagreb Croatia; ^2^ Department of Psychology Royal Holloway University of London Egham UK

**Keywords:** cross cultural validation, face memory, face recognition, famous face test

## Abstract

Familiar‐face tests are crucial to understanding face processing abilities but are culturally brittle. We investigated whether the Famous Faces Test (FFT) is valid and age‐robust in familiar‐face recognition in a non‐English‐speaking context. In two studies, Croatian younger and older adults completed a face recognition battery (FFT, CFMT, PI‐20), with potential developmental prosopagnosics reviewed via broader testing (OFMT, GFMT‐2). Wide inter‐individual variation in FFT did not show group differences in familiarity‐controlled accuracy. CFMT scores were comparable across ages, with moderate FFT correlations in both samples. PI‐20 showed small negative associations with FFT, indicating limited convergence between self‐report and objective performance. In Study/3, potential prosopagnosics performed worse than controls across all measures, with large effect sizes. These findings support culturally adaptable use of FFT for assessing familiar‐face recognition. The test is age‐appropriate in accuracy, relates meaningfully to unfamiliar‐face memory, and, used alongside perception and matching measures, helps identify individuals warranting detailed diagnostic assessment.

## INTRODUCTION

The ability to recognize and remember faces is one of the fundamental aspects of human social interaction, significantly impacting personal relationships, societal functioning, and playing an important role in social communication by allowing individuals to identify familiar people, interpret emotions, and develop social bonds (Young & Burton, 2018).

Understanding the multifaceted nature of human face perception has recently shifted in the direction of studying individual differences (Dunn et al., 2020; Stantić et al., 2021), giving rise to the development of a number of tests that can capture individual differences in typical populations. While most people have average face recognition skills, the literature recognizes a broad spectrum of abilities, ranging from developmental prosopagnosia (DP) to super recognition (SR). DP is a condition characterized by severe difficulties in recognizing faces despite normal vision and intelligence (Duchaine & Nakayama, 2006), which affects approximately 2%–3% of the population and is believed to result from atypical development of face‐processing networks rather than acquired brain damage (Bowles et al., 2009; Kennerknecht et al., 2006). Super recognition, on the other hand, shows exceptional face recognition abilities (Russell et al., 2009) and often excels at identifying individuals even from low‐quality or altered images.

A spirited debate in the field has been ongoing regarding the appropriate ways to diagnose developmental prosopagnosia (Bowles et al., 2009; Duchaine & Nakayama, 2006) and super recognition (Dunn et al., 2020; Russell et al., 2009) to ensure that the populations studied across different groups are sufficiently similar to make comparisons across studies worthwhile.

Arguments have been made that reliance on arbitrary cut‐offs can hinder comparability across studies and highlighted important caveats about using different diagnostic approaches and methodological variability across laboratories (see review by Manippa et al., 2023). Frameworks suggesting increased precision, inter‐laboratory consistency, and multi‐test approaches in diagnosing super recognizers have been suggested (Ramon, 2021) to alleviate these concerns. Recent work has highlighted continued challenges in both conceptually and methodologically defining developmental prosopagnosia, as well as setting appropriate diagnostic frameworks (Nørkær et al., 2024).

Largely, this debate has been focused on the groups in English‐speaking countries, where a number of standard measures have been deployed for this purpose in the last decade. Standardly, it is agreed that participants ought to score poorly on more than one measure to be classified as prosopagnosic (Murray & Bate 2020; Dalrymple & Palermo, 2016). Some tests, for instance the Cambridge Face Memory Test, have been deployed successfully across countries (McKone et al., 2011, 2012), requiring only translation of the instructions, as the cognitive processes they tap are culturally agnostic.

In non‐English speaking cultures, some tests are harder to use because their design *can make it more* difficult to align with local norms. Frequently used in identifying prosopagnosia is the Famous Faces Test (FFT, Duchaine et al., 2007), a neuropsychological assessment tool designed to determine how well people can recognize familiar identities of famous people (Hodges et al., 1993). The FFT is a theoretically robust test, used widely in both diagnostic and non‐diagnostic research settings. It is also ecologically valid, as it mirrors the types of familiar‐face recognition tasks people encounter in everyday life, for example, recognizing actors on television or identifying acquaintances in a natural environment (Bowles et al., 2009; Duchaine et al., 2007). However, it remains practically challenging to deploy due to cultural specificity. Individuals who are famous in one country may be substantially less well known in another; similarly, figures well known in one age group might be unfamiliar to another (Bowles et al., 2009). These limitations underscore the need for cultural validations of the FFT ahead of deployment in clinical settings for diagnostic purposes.

These adaptations have been administered in some contexts. For example, the Italian Famous Face Test (IT‐FFT) was recently developed to assess famous face recognition within the Italian population, providing normative data for both typical and clinical populations (Ventura et al., 2024). Similarly, Dutch researchers have recently developed a handful of versions of the test of a similar format using faces familiar to the target population (van den Elzen et al., 2023). Researchers in Portugal (Lima et al., 2021) and Greece (Proios et al., 2007) have also developed versions of the test appropriate for use in those populations, largely featuring familiar faces in their respective countries.

In addition to the international adaptations, multiple versions of the English test have been made over time, reflecting the changing cultural milieu. Several versions of the FFT have been created in English to accommodate cultural changes in celebrity familiarity (Duchaine et al., 2007; Russell et al., 2009). There are, of course, inherent limitations in famous face selection: changes in the popularity of individuals over time and differences in what various age groups perceive as “famous”. It is therefore necessary to validate that instruments like the FFT can be used across cultures without losing diagnostic sensitivity. Since memory and social engagement tend to change with age (Boutet et al., 2015; Stantić et al., 2021), older adults may know fewer contemporary celebrities than younger adults, potentially making such tests more complex for them.

The purpose of this study, therefore, is to determine the appropriateness of using the Famous Faces Test in the Croatian population for purposes of prosopagnosia diagnostics. The study will, in a series of experiments, test whether deployment of this test in two distinct groups – younger and older adults – yields results comparable to previously observed findings, as well as whether any age‐related differences in recognition performance arise.

Additionally, since the ultimate aim of this effort is to determine whether the FFT can be used in the Croatian population for developmental prosopagnosia screening, a third experiment will examine the performance of atypically poor performers (potential developmental prosopagnosics) to determine how FFT performance predicts performance captured by a wider battery of tests (CFMT, Duchaine & Nakayama, 2006; OFMT, Stantić et al., 2021; GFMT2, White et al., 2022) as well as self‐report measures (PI‐20; Shah et al., 2015). This will allow us to determine whether the use of FFT, alongside other standard measures, and in comparison with existing norms for the tests, allows for appropriate delineation of atypical performance in the population.

## METHODOLOGY

### Procedure – studies 1 and 2

Participants completed the college‐age version of the well‐established Famous Faces Task (FFT), the Cambridge Face Memory Test (CFMT, Duchaine & Nakayama, 2006), and the 20‐Item Prosopagnosia Index (PI‐20, Shah et al., 2015). All tasks were completed using the online behavioural platform, Gorilla.sc. Informed consent was obtained from all participants. Data acquisition took place between January and August 2024.

The ordering of tasks was done pseudorandomly in order to account for metacognitive priming. The CFMT and FFT were counterbalanced in order across participants, with half of the participants completing the CFMT first, followed by the FFT, and the other half administered tasks in the opposite order. The PI‐20 was always administered after both experimental tasks to prevent metacognitive priming or self‐reflection effects from influencing performance on the CFMT or FFT.

### Tasks

#### Cambridge Face Memory Test (CFMT, Duchaine & Nakayama, 2006)

The CFMT is a widely deployed and commonly used test of face recognition that focuses on measuring participants' unfamiliar face memory ability. It was originally developed to distinguish typical from atypical perceivers and is widely used as one of the tests underlying diagnostic criteria for developmental prosopagnosia.

Participants first learn six target faces and are then tested in three‐alternative forced‐choice trials. Each trial includes two distractor images and one image of a learned target identity. The test is divided into three stages of increasing difficulty: the first stage includes 18 test trials with no changes in viewpoint or lighting; the second stage includes 30 trials with changes in viewpoint and lighting; the final stage includes 24 trials with both changes in viewpoint and lighting and the addition of Gaussian visual noise. The maximum possible score is 72.

#### 20‐Item Prosopagnosia Index (PI‐20, Shah et al., 2015)

The PI‐20 is a self‐report measure of face recognition ability, comprising 20 items where participants rate on a Likert scale (1 = strongly disagree, 5 = strongly agree) assessing face recognition difficulties in everyday life (e.g., “I have always had a bad memory for faces”). Five items are reverse scored, the total score is calculated by summing the scores from all items. The PI‐20 can be used as a primary screening tool to identify individuals with difficulties in face recognition and can be used in conjunction with the CFMT to provide an initial indication of individuals who may require further testing for developmental prosopagnosia. The maximum possible score is 100.

#### Famous Faces Test (FFT, Duchaine et al., 2007)

The Famous Faces Test (FFT) is a widely used measure of familiar face recognition, which assesses participants' ability to recognize famous faces. The faces included in the test are well‐known politicians, athletes, actors, actresses, and other public figures. Fifty‐five context‐free faces are presented, and participants are asked to enter either a name or a uniquely identifying detail about the person presented.

In the first phase, participants identify the famous individuals from black‐and‐white images by typing in their name or other uniquely identifying information. In the second phase, participants are given a list of names of people they were presented with during the task and asked to indicate whether they are familiar with them. Accuracy is calculated only for identities marked as familiar, so the total number of trials varies between participants.

The Famous Faces Test‐College Age (FFT) was minimally adapted for use in Croatian populations. The test instructions were translated to Croatian by licensed psychologists, and instructions were given to participants that either a name or a unique identifying detail could be given to accurately recognize an identity. All 55 identities from the original version of the test were retained for a Croatian audience.

### Inclusion and exclusion criteria

Across both studies, participants were excluded if they met one or more of the following criteria:

1. Experienced technical difficulties that prevented finishing all parts of the task.
2. Reported current or recent use of psychotropic medication.
3. Were under 18 years old.
4. Did not have normal or corrected‐to‐normal vision.

### Sample size reasoning

Sample size decisions for all three studies were driven by a combination of practical constraints and existing face‐recognition literature.

For Studies 1 and 2, our recruitment target was 100 participants per group, which corresponds to sample sizes commonly used in individual‐differences work. Post hoc power analyses were conducted in G*Power and showed that with *N* = 110 and two‐tailed *α* set at .05, we had 90% power to detect medium sized correlations (*r* = .3).

In Study 3, the size of the follow‐up group was necessarily limited by the number of participants who met our screening criteria in Studies 1 and 2 and agreed to be additionally tested. Because of the small ultimate sample, group analyses are underpowered and only available in the Appendix S1. Our primary analysis is instead a single case dissociation and deficit review. We therefore report a sensitivity analysis rather than a post hoc power analysis. With *N* = 16 controls and one‐tailed *α* set at .05, the Crawford modified *t*‐test is sensitive to case deviations of approximately 1.8 SD from the control mean.

## EXPERIMENT 1: YOUNG ADULTS

Experiment 1 aimed to determine the validity and reliability of using the Famous Faces Test in the Croatian student (college‐aged) population. The primary aim of the study was to determine the performance range and assess the measure's usefulness as a test of individual differences for administration in assessment of possible developmental prosopagnosia. In addition, the performance on the test was compared to the existing objective computer‐based measures (CFMT, Duchaine & Nakayama, 2006) and self‐report measures (PI‐20, Shah et al., 2015).

### Participants

In Experiment 1, 111 college students (88 female, 23 male; average age: 20.9 years, SD: 1.90 years) took part in exchange for course credit. Participants completed the tasks online via Gorilla.sc testing platform. The demographic questionnaire was completed first, followed by three computer‐based tasks, presented in a random order.

### Results

CFMT: The scores on the CFMT ranged from 34 to 71, with a mean of 55.91 and SD of 9.19. This included two participants who scored lower than the commonly used cutoff of 42, who were investigated further below in the Multivariate scoring section.

PI‐20: The reported scores on the PI‐20 ranged from 25 to 69, with a mean reported score of 41.01 and SD of 11.26. There were two participants who scored over the threshold of 65, indicative of moderate everyday difficulties, and these participants are investigated further below in the section Multivariate scoring.

FFT: Participants reported being familiar with *M* = 41.59 identities (range 11–55, SD = 8.65). Taking into account only the identities that were marked as familiar, participants accurately recognized an average of 79.06% of identities, with individual accuracies ranging from 15.38% to 100% (SD = 15.38%). Four participants scored below 50% and are investigated further below in the section Multivariate scoring.

Relationships between tests were also noted. As expected, the correlation between CFMT and FFT was moderate at *r* = .41, *p* < .001.

**FIGURE 1:**
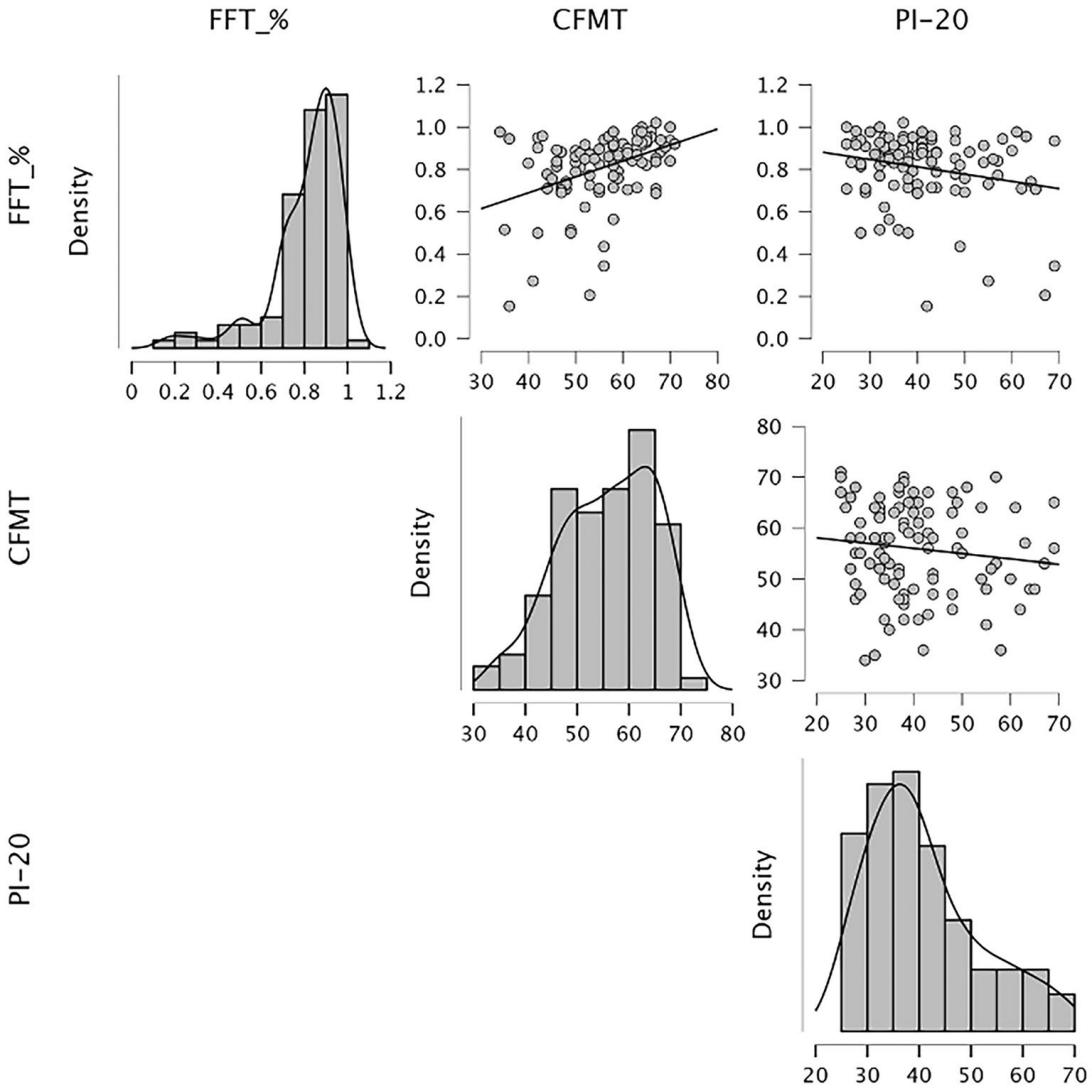
Scatterplot matrix showing distributions and bivariate relationships among measures of face recognition ability in Experiment 1. The diagonal panels show histograms with density estimates for each measure: FFT_% (proportion of familiar famous faces correctly identified), CFMT (Cambridge Face Memory Test, raw score out of 72), and PI‐20 (20‐Item Prosopagnosia Index, higher scores = more self‐reported difficulty). Off‐diagonal panels display scatterplots with least‐squares regression lines, illustrating moderate positive associations between CFMT and FFT_% and negative associations between PI‐20 and both FFT_% and CFMT.

Relationships with PI‐20 were notably different – the correlation between FFT and PI‐20 was −.22 (*p* < .05), indicating that the lower PI‐20 scores, and lower self‐reported difficulties with face perception, were associated with higher accuracy in recognizing famous faces. However, the relationship between PI‐20 and CFMT was not significant (*r* = −.13, *p* = .22), indicating no predictive relationship between the two tests (Figure 1).

## EXPERIMENT 2: OLDER ADULTS

The primary aim of Experiment 2 was to determine the validity of using the Famous Faces Test in non‐college populations in Croatia. Given that it was originally developed to be culturally closer to a college‐student population in the US, the test needed validation in a substantially different culture and age group before being deployed in the assessment of potential difficulties in face perception, such as developmental prosopagnosia. As in Experiment 1, the performance on the test was compared to the existing measures, CFMT and PI‐20.

### Participants

110 participants (82 women, 28 men; average age: 46.85 years, SD: 10.08 years) took part in the experiment in exchange for being entered into a bookstore voucher draw.

### Results

*CFMT*: The scores ranged from 34 to 72 (*M* = 54.96; SD = 10.41). There were five participants who scored below the standardly used threshold of 42, who are further investigated below in the section Multivariate scoring.

*PI‐20*: The scores ranged from 20 to 85 (*M* = 45.68, SD = 14.47). Five participants scored above the threshold of 65 and are further investigated below in the section Multivariate scoring.

*FFT*: Participants reported being familiar between 19 and 55 of the presented identities (*M* = 45.08, SD = 8.16). Taking into account only the identities marked as familiar, participants accurately recognized an average of 75.26% of identities (range = 3.84%–98.15%, SD = 18.27%). Four participants scored below 50% and are further investigated below in the section Multivariate scoring.

Relationships across scores were largely in line with expectations from literature. The correlation between CFMT and FFT was moderate at *r* = .343, *p* < .001.

**FIGURE 2:**
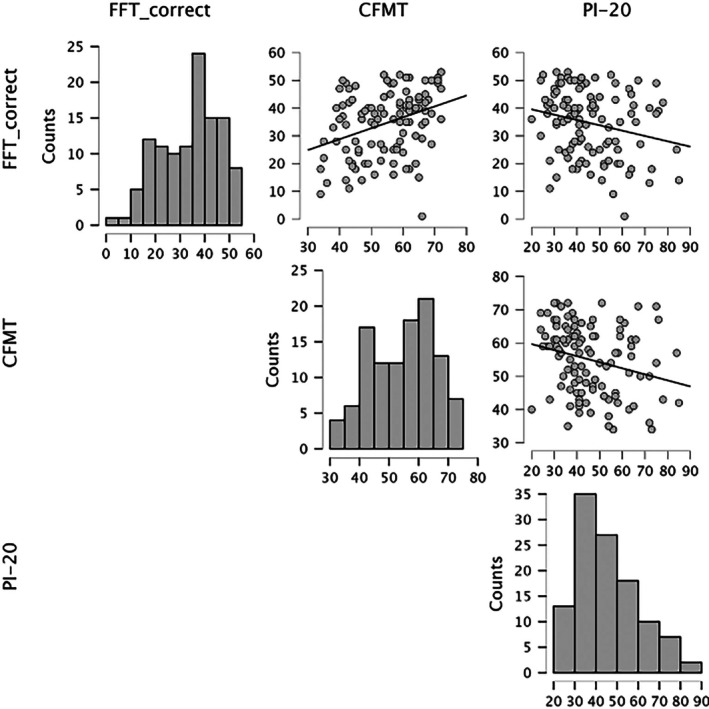
Scatterplot matrix showing distributions and bivariate relationships among measures of face recognition ability in Experiment 2 (*N* = 110). The diagonal panels display histograms for each measure: FFT_correct (raw number of familiar famous faces correctly identified, maximum = 55), CFMT (Cambridge Face Memory Test, score out of 72), and PI‐20 (20‐Item Prosopagnosia Index, higher scores = more self‐reported difficulty). Off‐diagonal panels show scatterplots with least‐squares regression lines.

Relationships with PI‐20 were somewhat different from Study 1. Both showed small negative correlations with FFT, *r* = −.23 (*p* = .02), and with CFMT, *r* = −.25 (*p* = .01). This indicates the overall direction of lower self‐reported difficulties with face perception is associated with higher accuracy in recognizing famous faces, as well as higher accuracy on the face memory measure (Figure 2).

### Analyses across experiments 1 and 2

The main aim of Experiment 2 was to test whether use of FFT in an adult Croatian population was appropriate. We hypothesized that, due to cultural and age differences between these populations, we may observe lower overall scores on the test in the older adult group, which would raise questions about the use of this tool in DP diagnostics across these groups. However, the data did not support this. While there was a significant difference between younger (*M* = 41.6, SD = 8.67) and older adults (*M* = 45.08, SD = 8.16), *t*(222) = 3.10, *p* = .002, *d* = .42, this difference was in the direction of older adults recognizing more identities than younger adults. This pattern most likely reflects cohort‐specific differences in exposure to media and the period during which particular celebrities were most prominent, rather than true age‐related differences in familiar‐face recognition ability. Importantly for the diagnostic application, however, there was no significant difference between younger (*M* = 79.1%, SD = 17.4%) and older adults (*M* = 75.3%, SD = 18.3%) in recognition accuracy on the FFT, *t*(222) = −1.59, *p* = .11, *d* = .21.

There were also no significant differences between the younger (*M* = 55.91, SD = 9.19) and older adults (*M* = 54.95, SD = 10.41) on the face memory scores measured using the CFMT, *t*(222) = −.72, *p* = .47, *d* = .10.

However, there was a significant difference in self‐reported difficulties with face perception in everyday life between younger (*M* = 41.01, SD = 11.26) and older (*M* = 45.68, SD = 14.49) adults, *t*(222) = 2.69, *p* = .01, *d* = .36. (Note that the Levene's test for equality of variances was significant *F*(1, 222) = 7.24, *p* = .01, indicating that the assumption of homogeneity of variances was violated). Therefore, the Welch *t*‐test, which does not assume equal variances, was used to compare group means on the PI‐20. For other variables, the standard Student's *t*‐test was used as there were no violations of the quality of variances. This is in line with previous literature showing that older adults are more likely to report, as well as experience, difficulties with face recognition (Boutet et al., 2015; Fry et al., 2023; Stantić et al., 2021).

To further examine the ability of self‐report functions to capture possible DP diagnoses, we examined the participants whose PI‐20 *z*‐scores were larger than 2, as well as those with raw scores higher than 65.

The first analysis resulted in nine participants, three of whom were flagged for further investigation after accounting for their scores on the FFT and the CFMT. The remaining six participants showed a typical pattern of CFMT and FFT results for their age group (with *z*‐scores ranging from −1.26 to 1.55 for the CFMT, and −.25 to .81 for the FFT, indicating a wide but typical range of scores for the remainder of the group). The second analysis also included participants who scored over 65 but did not reach the threshold of *z*‐scores greater than 2, which represented another three participants. None of these participants showed any other indication of potentially abnormal face processing on the CFMT or FFT.

### Multivariate scoring

Participant scores were analysed for deviations from the mean to identify outliers with poor perception (see Bowles et al., 2009). Across Experiments 1 and 2, participants were flagged if they met two or more criteria for developmental prosopagnosia.

For potential prosopagnosia, participants with CFMT scores <42, PI‐20 scores >65, or FFT scores <50% were identified. In line with commonly used diagnostic frameworks for developmental prosopagnosia (Dalrymple & Palermo, 2016; Nørkær et al., 2024), impaired performance on at least two face‐processing tests is required for prosopagnosic classification. In our samples, 4 participants in Study 1 and 6 participants in Study 2 met those criteria. They were flagged for further testing using a richer battery of face recognition tests in Study 3. Descriptive scores and *z*‐scores of those participants are available in Table 1 below. Note that one of the measures taken into account here is PI‐20, the self‐report questionnaire, which has been shown to have variable reliability in different populations (Bobak et al., 2019, Estudillo & Wong, 2021, see also critique in review by Nørkær et al., 2024), but most laboratories deploy a combination of objective and self‐report measures (Bate & Tree, 2017) so our strategy here sought to include more potentially prosopagnosic participants rather than excluding them at this stage (Arizpe et al., 2019, also see Burns et al., 2023 for discussion of this issue).

**TABLE 1:** Scores of potentially prosopagnosic participants identified from Experiments 1 and 2.

Participant ID	FFT recognized	FFT_%	CFMT	PI‐20
Exp1/1	34	20.59	53	67
Exp1/2	32	34.38	56	69
Exp1/3	34	50	42	38
Exp1/4	26	15.38	36	42
Exp2/1	35	40	42	85
Exp2/2	48	37.5	34	73
Exp2/3	49	83.67	41	65
Exp2/4	54	50	50	68
Exp2/5	24	37.5	34	56
Exp2/6	20	65	36	72

*Note*: Participants are identified simply by their order of participation in each applicable Experiment. FFT Recognized refers to the number of recognized identities, whereas FFT_% indicates a percentage of correctly identified identities from the identities marked as known to each participant individual. CFMT represents raw Cambridge Face Memory Test scores, and the PI‐20 represents raw scores on the 20‐Item Prosopagnosia Index.

We emphasize, therefore, that this does not constitute a clinical diagnosis of DP. Full diagnostic classification requires a multi‐test battery that exceeds the scope of Studies 1 and 2.

Participants show notably different profiles. Some reported few perceived difficulties with everyday face perception (e.g., Exp1/4) but show objective scores that deviate significantly from the typical population. Other participants (e.g., Exp1/2) show impaired performance mainly on the FFT and report a high level of perceived difficulties with everyday recognition, but their CFMT scores are similar to the group mean.

## EXPERIMENT 3: FOLLOW UP OF POTENTIAL DEVELOPMENTAL PROSOPAGNOSICS

Participants identified in the Multivariate scoring section were invited for a follow‐up study to further investigate their face processing ability using a more targeted and richer battery of tests.

As discussed previously, the ability to recognize and process faces significantly varies among individuals. While most people show face recognition abilities within a typical range, there are some individuals who belong to extremes of the spectrum – those with prosopagnosia (face blindness) and those with exceptional face recognition abilities, known as super‐recognizers (SRs) (Duchaine & Nakayama, 2006; Russell et al., 2009).

The primary aim of Experiment 3 was to gain a richer understanding of the possibly impaired performance in the potentially prosopagnosic participants identified in Experiments 1 and 2 through screening of the general population. An additional battery of tests was administered on follow‐up, and an age‐ and gender‐matched control group was recruited to provide an appropriate comparative baseline. The additional tests included measures of face perception, face matching, and face memory, all of which have been shown to independently contribute to face recognition ability (Stantić et al., 2022). The importance of using multi‐test batteries to assess face processing deficits has been well documented (Bowles et al., 2009; White et al., 2022). Our goal in this experiment was to assess whether the atypical face processing abilities observed in the earlier screening stages could be further described using more in‐depth and validated cognitive measures. Furthermore, with this approach, we wanted to distinguish deficits in face perception from memory impairments. While face perception deficits involve difficulties in differentiating between visually similar individuals, memory deficits relate to the ability to retain and retrieve previously learned facial information (White et al., 2022). This distinction is essential, as impairments in one domain do not necessarily imply deficits in the other (Stantic et al., 2022; Stantić et al., 2023), and a more nuanced understanding of these cognitive processes may aid in refining diagnostic criteria for conditions like developmental prosopagnosia (DP).

### Procedure

Participants completed the long version of the Oxford Face Matching Test (OFMT, Stantić et al., 2022), the updated version of the Glasgow Face Matching Test (GFMT2, White et al., 2022). All tasks were completed using the online behavioural testing platform Gorilla.sc. Informed consent was obtained from all participants. Data acquisition took place between September and December 2024. Each participant completed the three tasks in a randomized order to mitigate order effects.

### Tasks

#### Oxford Face Matching Test (Stantić et al., 2022)

The OFMT is a recently developed test that measures individual differences in face perception across the full spectrum of performance, from those with poor face recognition ability to those with superior face recognition ability.

Participants are shown 200 pairs of faces and asked to judge whether the faces belong to the same person or different individuals. The test uses images that vary in their difficulty based on facial similarity, as assessed by facial recognition algorithms. Participants view each pair of faces side by side for 1600 ms and are required to judge the similarity between the faces and determine whether they are of the same individual or not. This task aims to assess face perception ability without relying on memory. An additional feature of the OFMT is that it allows for objective comparison of participants' responses to the algorithms, avoiding bias to neurotypical norms. This allows a dissociation of face perception from face matching abilities, which has been shown to be sensitive to atypical performance in autism and developmental prosopagnosia (Stantić et al., 2022; Stantić et al., 2023).

#### Glasgow Face Matching Test 2 (White et al., 2022)

The GFMT2‐S is a recently developed test of unfamiliar face matching ability, assessing participants' ability to match face images without relying on memory. It is expected to be a more sensitive measure than the original GFMT, useful in studying face recognition impairments like prosopagnosia.

Participants in the GFMT2‐S view pairs of face images and decide whether they show the same person or different people. The task includes 80 items in total, featuring images that vary in head angle, pose, facial expression, and subject‐to‐camera distance, increasing the ecological validity of the task.

### Participants

Twenty‐four total participants took part. Eight participants (five female, three male, average age: 38.4 years, SD: 18.6 years) responded to the follow‐up and took part in the experiment in exchange for a bookstore voucher. A control group of 16 participants (10 female, six male, average age: 38.0 years, SD: 17.5 years) was recruited to allow direct comparison to the experimental sample.

### Results

Tests of normality (Shapiro–Wilk) indicated that all variables were normally distributed in both groups (*p* > .05), justifying the use of parametric *t*‐tests. Levene's tests for equality of variances showed no significant violations (*p* > .05), and variance ratios were within acceptable ranges, supporting the assumption of homogeneity of variance.

To assess the relationships between different facets of face processing ability, Pearson correlation coefficients were calculated, although the sample size for these correlations was notably small. All correlations were statistically significant.

OFMT scores were significantly correlated with FFT (*r* = .83, *p* < .001), indicating that individuals with better perceptual face matching ability also performed better on familiar face recognition. OFMT scores also correlated significantly with CFMT scores (*r* = .69, *p* < .001), suggesting that there is a shared variance between perceptual discrimination and face memory ability. Finally, the two face‐matching tasks, OFMT and GFMT2, correlated significantly (*r* = .68, *p* < .001).

FFT also correlated strongly with GFMT2 (*r* = .61, *p* < .01) and CFMT (*r* = .71, *p* < .001), indicating that better recognition of famous faces is associated with both perceptual discrimination performance as well as face memory performance.

Overall, lower objective scores on all face‐processing tasks accompany higher self‐reported difficulty in the PI‐20 (higher scores indicate more perceived difficulties). The PI‐20 showed moderate to strong negative correlations with all objective face processing measures: OFMT (*r* = −.66, *p* < .001), GFMT2 (*r* = −.65, *p* < .001), FFT (*r* = −.66, *p* < .001), and CFMT (*r* = −.53, *p* < .01). Thus, participants who considered themselves better at recognizing faces (lower PI‐20 scores) also objectively performed better on computer‐based tasks of perceptual discrimination and face memory, supporting the convergent validity of the full test battery.

### Case study analyses

**FIGURE 3:**
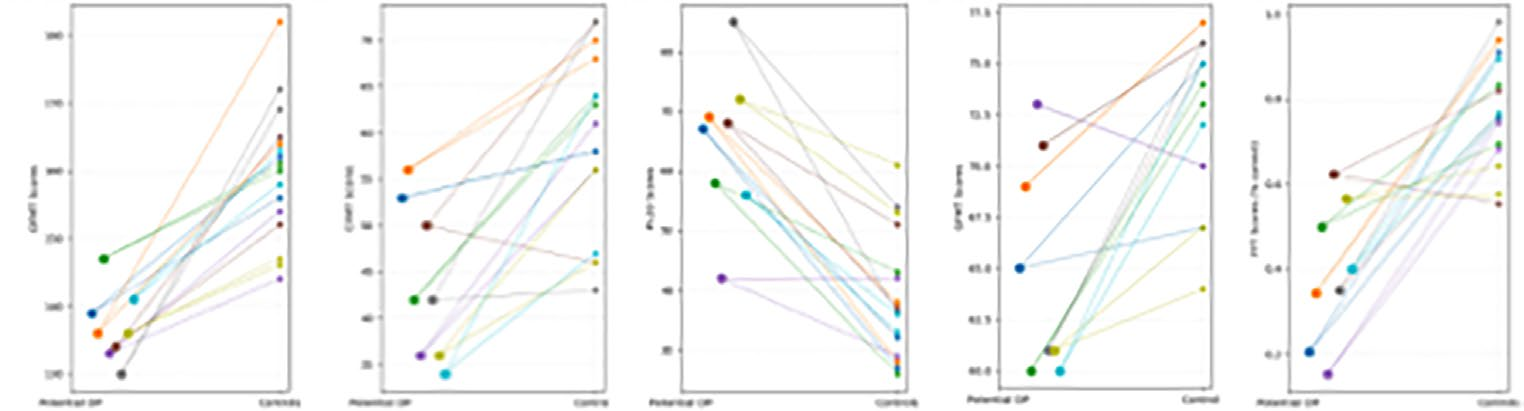
Paired comparisons of potential developmental prosopagnosic participants and their matched controls across face processing and self‐report measures. Each line represents a matched pair, with scores plotted for (from left to right): (1) Oxford Face Matching Test (OFMT), (2) Cambridge Face Memory Test (CFMT), (3) PI‐20, (4) Glasgow Face Matching Test (GFMT2), and (5) Famous Faces Test (FFT; proportion correct).

While group comparisons can offer a useful insight into generalized performance of two potentially separate portions of the population, the small size of this sample and the range of ages of potentially DP participants necessitate a more nuanced analytical approach. Careful age and gender matching was performed to a 2:1 ratio such that each potential DP participant was matched to two control participants of the same age and gender. Here we include a case‐by‐case descriptive analysis of those pairings. A visual inspection of the scores shows clearly that the differences observed at the group level can also be observed at an individual level for almost every patient + matched control. These differences are most prominent for the OFMT scores, where the biggest group difference was also observed. See Figure 3 for more details.

### Single case deficit and dissociation analyses

Given the limited power for inferences at group level due to modest samples, we conducted additional single‐case methods to characterize individual profiles comparing each suspected prosopagnosic participant to the full control sample while statistically controlling for age. We used an existing framework for describing single cases (Crawford et al., 2011) and looked at whether each individual's scores were significantly lower than expected given their age. In including age as a covariate, we are able to test whether differences between potential prosopagnosic participants and controls remain after partialing out age‐related variance on performance. We additionally tested for dissociation between different measures applied to age‐adjusted scores to understand behavioural profiles across familiar identification, face matching, and face memory.

#### Deficits (age‐covariate single‐case tests)

After controlling for age, deficits were consistently observed on FFT (6/8 cases), OFMT (6/8 cases), and PI‐20 (6/8 cases). There were slightly fewer deficits observed on GFMT2‐S (5/8 cases) and CFMT (3/8 cases). Full case‐by‐case results (test statistic, *p*‐value, and estimates of abnormality) are reported in Table 2.

**TABLE 2:** Single‐case deficit analyses controlling for age (Exp. 3).

Participant ID	CFMT			FFT			OFMT			GFMT2			PI20		
	Raw	*p*	Abn (%)	% correct	*p*	Abn (%)	Raw	*p*	Abn (%)	Raw	*p*	Abn (%)	Raw	*p*	Abn (%)
Exp1/1	53	.17	16.73	20.6	<.01*	.02	139	.02*	2.41	65	.03*	3.15	67	<.01*	.10
Exp1/2	56	.25	25.24	34.4	<.01*	.13	136	.01*	1.41	69	.15	14.85	69	<.01*	.06
Exp1/3	42	.02*	2.47	50.0	.01*	1.34	147	.09	9.03	60	<.01*	.33	58	.01*	.72
Exp1/4	36	.01*	.72	15.4	<.01*	<.01	133	.01*	.81	73	.44	43.77	42	.16	15.60
Exp2/1	50	.26	26.03	62.3	.18	18.27	134	.02*	1.96	71	.40	40.30	68	.01*	.87
Exp2/2	42	.05	5.44	35.1	<.01*	.30	130	.01*	.71	61	.01*	.78	85	<.01*	<.01
Exp2/3	36	.06	5.56	56.5	.17	16.79	136	.05*	4.78	61	.02*	1.90	72	.02*	1.64
Exp2/4	34	.02*	2.36	40.0	.01*	1.36	141	.08	7.75	60	.01*	.82	56	.15	15.10

*Note*: Raw scores are shown for each potential DP case. *p*‐values are one‐tailed in the direction of expected impairment (lower scores for tasks; higher scores for PI‐20), with age included as a covariate using the control sample (*n* = 16). Abn (%) indicates abnormality percentage (the estimated percentage of the control population expected to obtain a score as extreme or more extreme in the impairment direction).

* Significance at *p* < .05 level.

#### Dissociations (age‐adjusted)

Dissociation tests indicated that some cases showed disproportionately poor performance on specific measures relative to others. In particular, three cases showed a significant dissociation between CFMT and FFT (with FFT relatively worse), two cases showed a dissociation between FFT and OFMT (with FFT relatively worse), and two cases showed a dissociation between OFMT and GFMT2‐S (with OFMT relatively worse). These dissociations suggest that impairment profiles are not uniform across tasks and are consistent with partially separable contributions of familiar identification, memory, and perceptual matching. Dissociation test statistics and abnormality estimates are reported in Table 3.

**TABLE 3:** Single‐case dissociation analyses controlling for age (Exp. 3).

Participant ID	CFMT and FFT				CFMT and OFMT				FFT and OFMT				OFMT and GFMT2			
	*t*	*p*	Abn (%)	Worse	*t*	*p*	Abn (%)	Worse	*t*	*p*	Abn (%)	Worse	*t*	*p*	Abn (%)	Worse
Exp1/1	4.38	<.01*	.03	FFT	1.12	.28	14.1	OFMT	3.21	.01*	.3	FFT	.17	.87	43.4	OFMT
Exp1/2	3.55	<.01*	.2	FFT	1.69	.11	5.6	OFMT	1.55	.14	7.1	FFT	1.61	.13	6.6	OFMT
Exp1/3	.39	.70	35.1	FFT	.71	.49	24.4	CFMT	1.36	.19	9.7	FFT	2.09	.05	2.7	GFMT2
Exp1/4	2.78	.01*	.7	FFT	.06	.95	47.7	CFMT	3.01	.01*	.4	FFT	3.02	.01*	.4	OFMT
Exp2/1	.33	.74	37.2	FFT	1.53	.15	7.3	OFMT	1.69	.11	5.6	OFMT	2.35	.03*	1.6	OFMT
Exp2/2	1.79	.09	4.7	FFT	1.02	.32	16.2	OFMT	.54	.60	30	FFT	.06	.96	47.8	OFMT
Exp2/3	.89	.39	19.3	CFMT	.09	.93	46.6	OFMT	1.06	.31	15.4	OFMT	.62	.54	27	GFMT2
Exp2/4	.36	.73	36.3	FFT	.65	.53	26.3	CFMT	1.24	.23	11.7	FFT	1.45	.17	8.4	GFMT2

*Note*: Each suspected DP case was compared to the control sample. Dissociations between task pairs were then tested using the Revised Standardized Difference Test on age‐adjusted values. *T*‐values refer to the *t*‐equivalent statistic for the discrepancy (df = 15), *p*‐values are two‐tailed. Abn (%) indicates the estimated percentage of the control population expected to show a discrepancy as large or larger in the observed direction; ‘Worse’ indicates the task that showed relatively poorer performance.

* Significance at *p* < .05 level.

## DISCUSSION

The primary aim of this study was to understand the performance of the Famous Faces Test in the Croatian population, given its prominent use in determining difficulties with face perception. Further to this primary research question, a distinction between younger and older adults was examined to see whether any differences arise in relation to age of participants, or if the test shows equivalent promise for use across the lifespan. Finally, its validity in capturing the poor performers in wide population‐level screening was examined by investigating the performance of screened potentially poor performers on a wider variety of established face measures.

Results indicate that the FFT shows an expectedly wide range of scores in the Croatian population. This is true both when examining the number of identities that were indicated as familiar, as well as the accuracy of recognition of those identities (Bowles et al., 2009; Dennett, McKone, Edwards, & Susilo, 2012; Dennett, McKone, Tavashmi, et al., 2012; Duchaine & Nakayama, 2006). While there were statistically significant differences in the number of identities indicated as familiar between younger and older adults, the direction of this relationship (older adults indicating more identities were familiar than younger adults) and numerical difference between the scores (41 vs. 45 identities) do not raise cause for concern in use of the FFT in either segment of the population. Most importantly, results indicate that there were no significant differences in accuracy of recognition between the groups once the number of recognized identities was accounted for. This indicates that the test has equivalent sensitivity to measure individual differences in these two groups. As a result, researchers looking to conduct wide range screening of the general population can be confident in using this measure. The ease of administration makes the FFT a good choice of measures to include in a prescreening battery.

Further, no significant differences arose in observed face memory between groups, and the range of scores observed is in line with previous research on healthy neurotypical adults (Fry et al., 2023; Stantić et al., 2021; Ventura et al., 2024). Given the focus on typical participants in this study, and relatively small differences in average age of the groups, we did not expect to observe an age effect using the CFMT (as has been reported with much older adults, Fry et al., 2023, but see age‐bucketed scores for indicative norms, Stantić et al., 2021). However, given the prominent role that CFMT plays in diagnosing prosopagnosia and existing literature indicating overlapping cognitive processes in these two tasks (Bowles et al., 2009; Dennett, McKone, Edwards, & Susilo, 2012; Dennett, McKone, Tavashmi, et al., 2012), we expected a significant positive relationship between scores on CFMT and FFT, which was observed. This indicates that individuals who show better recognition of familiar famous faces also show better memory for unfamiliar faces in this sample. The moderate size of this relationship is, as previously argued in the literature, likely a result of these two tests tapping into different specific components of face recognition processes, which are overlapping but not entirely shared (Dennett, McKone, Edwards, & Susilo, 2012; Dennett, McKone, Tavashmi, et al., 2012; Wilmer, 2017). Further variance is likely explained by use of familiar and unfamiliar faces, which are known to engage distinct cognitive (Burton et al., 2011; Gobbini & Haxby, 2007) and neural (Haxby et al., 2000; Natu & O'Toole, 2011) mechanisms.

One interesting finding was that the number of identities marked as familiar was higher for older than younger adults. One possible explanation lies in a methodological choice in the development of the original FFT College‐Age. This version of the test was developed in response to a change in familiarity with identities from the original FFT about 15 years ago (Duchaine et al., 2007), making the older population in our current sample more age‐aligned with many of the figures used in the test. This finding, however, indicates that the identities appropriate for testing individual differences in familiar face recognition are continually changing and that any test of famous faces likely needs regular updating to stay relevant. Of course, true measures of familiar face recognition would likely include idiosyncratic design for each participant, featuring faces that are familiar to them; due to the tedious methodological nature of such an approach, this is not commonly done (though see Nijhof et al., 2023).

The relationship between PI‐20, a self‐report measure of one's own face recognition ability, and the objective tests FFT and CFMT varied between the two samples. In the younger sample, we observed a small significant negative relationship between self‐reported scores and the ability to recognize famous faces, but no significant relationship between self‐reported scores and the ability to memorize faces. In the older group, both tests showed a small negative relationship. This pattern of results indicates that, overall, higher self‐reported difficulties with face perception are in fact associated with lower accuracy of face processing ability, both in the recognition and memory domains.

Further, a review of participants who indicated they thought they had poor face perception compared to their peers showed only a very weak relationship between this belief and objective test scores, with only 3 out of 12 participants meeting criteria for further evaluation. Although the present study was not designed to identify super‐recognizers, the wide performance range observed on the FFT suggests that future work could also examine its utility at the upper end of the ability spectrum, where high accuracy on familiar‐face recognition tasks may provide meaningful complementary information to existing SR screening batteries.

These results are in line with previously somewhat inconsistent findings, which have indicated that typical adults likely have only a small‐to‐moderate insight into their ability (Bobak et al., 2018; Bowles et al., 2009; Stantić et al., 2021). This further reinforces the questions that have been posed in the literature about the suitability of using self‐report measures in diagnosing performance extremes. Based on the results we show here, a more suitable approach includes a shorter battery of engaging tests alongside a self‐report measure as a first line of screening, followed by a more detailed testing procedure. Future diagnostic work will also need to include measures of divergent validity, such as non‐face memory or object‐recognition tasks (e.g., the Cambridge Car Memory Test; Dennett, McKone, Edwards, & Susilo, 2012, Dennett, McKone, Tavashmi, et al., 2012), to ensure that performance differences on the FFT reflect face‐specific processes rather than broader visual perception ability. Another notable limitation of the present work is the gender imbalance in our samples, which may constrain the generalizability of our findings. Although we did not observe gender effects, future research with more gender‐balanced samples will more appropriately determine whether own‐gender effects on face‐recognition performance replicate in the Croatian population.

It was not of primary interest to this research group to examine individual differences at the top end of the spectrum, in the ‘super recognizer’ groups (Russell et al., 2009). However, the wide range of performance observed on the Croatian FFT, and no notable ceiling effects in either study, shows that utilization of this form of the test may, in conjunction with other appropriate measures (CFMT+, OFMT‐Long Form, GFMT2) be a useful method of both selecting and classifying super recognizer participants.

The pattern observed in Experiment 3, where the potential DP group showed impairments in both perceptual matching (OFMT, GFMT2) and memory‐based recognition (CFMT, FFT), is particularly informative. Recent work shows that deficits in face perception and face memory can dissociate meaningfully at the individual level (Stantić et al., 2022). This indicates that not all atypical face‐processing profiles share a common underlying cause. The fact that individuals flagged in our screening displayed reduced performance across both domains suggests that the subset captured by this battery may reflect a more globally impaired face‐processing profile, rather than a selective deficit in either perception or memory. Even though our sample is small, this shows that individuals with developmental prosopagnosia often demonstrate multi‐domain impairments in face processing.

Importantly, while our study identifies individuals showing atypical performance patterns, these should not be interpreted as confirmed cases of developmental prosopagnosia. Diagnosis requires convergence across multiple independent tests, ideally including both memory and perceptual measures. Our sample captures the first medium‐scale Croatian screening efforts, where no clinically confirmed DP cases have been reported (which is the ultimate aim of our work). Additionally, because several correlational analyses were conducted, particularly in Experiment 3, we note that these results were not corrected for multiple comparisons and larger scale testing (Stantic et al., 2022) should be used for more reliable measures of relationships between tasks. The small sample size in Experiment 3 reduces the generalizability of these findings and limits the power to reliably detect relationships across tasks, which can better be accomplished with large scale testing of diagnosed prosopagnosics (Stantić et al., 2022). Conclusions from this experiment should therefore be interpreted cautiously, and future studies in Croatia will require larger, more diverse samples to strengthen diagnostic inference. Indeed, one of the directions that future research will need to investigate is the minimal battery of tests needed for a diagnosis of DP (or SR) status. We are obviously able to derive much finer estimates of any individual's face perception ability when using a multitude of measures (Bowles et al., 2009; Stantic et al., 2022; White et al., 2022) and more sophisticated analyses, such as Balanced Integration Scores (Lowes et al., 2024) to determine one's level of ability. However, the practicality of the long testing sessions this normally entails limits the use of such an approach. This is particularly true for researchers who are seeking to determine the prevalence of particular traits in the overall population, or screening participants who do not have the metacognitive awareness of their deficits, for instance participants with high autistic traits (Shah et al., 2015; Stantić et al., 2023). It is, therefore, important to determine which measures can serve as a first‐line set of tests that can reliably, with low levels of incorrect rejections, screen participants to be investigated further.

Based on our Experiment 3 results, a two‐tiered testing approach may be the most efficient framework for population‐level screening. Tier 1 would include brief, sensitive measures such as the CFMT (Duchaine & Nakayama, 2006) and either the OFMT (Stantić et al., 2022) or GFMT2 (White et al., 2022), which are effective at detecting atypical performance across the full range of neurotypical variability. Testing an individual case against a large set of controls (with age as a covariate) would indicate abnormality on those measures, clarifying the necessity of further testing. Tier 2 would involve more intensive diagnostic tools (e.g., CFMT+, Murray & Bate, 2020; Russell et al., 2009; UNSW Face Test, Dunn et al., 2020; or extended OFMT version, Stantić et al., 2022) to provide a more detailed characterization of individuals flagged during initial screening. For countries where prosopagnosia screening is yet in its nascent phases, this could present a reasonably affordable yet scientifically rigorous way to screen a large population for deficits.

## AUTHOR CONTRIBUTIONS

**Maja Kolanović:** Conceptualization; writing – original draft; writing – review and editing; project administration; data curation; formal analysis. **Mirta Stantić:** Conceptualization; investigation; funding acquisition; writing – original draft; methodology; visualization; writing – review and editing; software; formal analysis; data curation; supervision; resources.

## FUNDING INFORMATION

This research was not funded via any named grants. We thank the Royal Holloway University of London for support of Mirta Stantic's work via her startup allowance.

## CONFLICT OF INTEREST STATEMENT

The authors, Dr. Mirta Stantic and Maja Kolanovic, MSc, declare that they have no known competing financial interests or personal relationships that could have appeared to influence the work reported in this paper.

## ETHICS STATEMENT

Ethical approval was granted by the University of Zagreb, Filozofski fakultet.

## PATIENT CONSENT

Participants in this research provided informed consent about their participation in the study, were able to withdraw at any point without providing a reason for doing so, and were provided a way of withdrawing their data from the study after its completion.

## Supporting information


Appendix S1.

## Data Availability

This study was not preregistered due to its exploratory nature. All experimental data will be made available fully anonymized via OSF: https://osf.io/v74cq. Authors will share their copyrighted materials or point any interested parties to appropriate contacts for materials they do not own copyright to.
