# Supplementary material for: Globally famous, locally recognized: Cross‐cultural validation of the use of Famous Faces Test in Croatia
Source: J Neuropsychol. 2026 Apr 17;20(2):468–86. doi: 10.1111/jnp.70047 (PMC13250373; doi:10.1111/jnp.70047)
Supplement: Supplementary file 1 — Appendix S1. [file JNP-20-468-s001.docx]

**Appendix S1 – exploratory analyses**

**Group Comparisons**

Given the modest sample which is likely underpowered to reliably detect differences across groups, correlational analyses here should be treated as exploratory. Independent sample t-tests showed statistically significant differences between the potential DPs and matched controls on all measures of face perception, face memory, and self-report questionnaires. Participants in the control group (M = 160, SD = 10) outperformed potential DPs (M = 137, SD = 5.29) on the Oxford Face Matching Test (OFMT), with a large effect size (t (22) = 5.96, *p* < .001, Cohen’s d = 2.58). A similar pattern was observed with the Glasgow Face Matching Test 2 (GFMT2) (t (22) = 4.00, *p* < .001, d = 1.73), with control participants (M = 72.8, SD = 4.02) scoring better than the potential DPs (M = 65, SD = 5.32).

On the Famous Faces Test (FFT_%), the potential DPs (M = .39, SD = .17) demonstrated significantly reduced ability of recognizing familiar faces compared to the control group (M = .78, SD = .13), t (22) = 6.29, *p* < .001, d = 2.72. Finally, the Cambridge Face Memory Test (CFMT) was also significantly lower in the experimental group (M = 43.6, SD = 8.42) than in the control group (M = 59, SD = 9.55), (t (22) = 3.86, *p* < .001, d = 1.67). Given that participants were selected based on their unusually low CFMT scores, this finding is expected. Self-reported face recognition difficulties, measured by the PI-20, were significantly higher in the potential DP group (M = 64.6, SD = 12.7; higher scores = more perceived difficulty) than in the control group (M = 39.1, SD = 10.7). Significant difference was found (t (22) = -5.17, *p* < .001, d = -2.24).

Figure 1 - Appendix. Group differences on all administered tasks between potential developmental prosopagnosia (potential DP) participants (in light blue) and controls (dark blue). Boxplots show median, interquartile range, and individual data points for (from left to right) with scores plotted for (from left to right): (1) Oxford Face Matching Test (OFMT), (2) Glasgow Face Matching Test (GFMT2), (3) Cambridge Face Memory Test (CFMT), (4) Famous Faces Test (FFT; proportion correct, and (5) 20-Item Prosopagnosia Index (PI-20).

Table 1. Scores of potentially prosopagnosic participants identified from Experiments 1 and 2. Participants are identified simply by their order of participation in each applicable Experiment. FFT Recognized refers to the number of recognized identities, whereas FFT_% indicates a percentage of correctly identified identities from the identities marked as known to each participant individual. CFMT represents raw Cambridge Face Memory Test scores, and the PI-20 represents raw scores on the 20-Item Prosopagnosia Index.

| **Participant ID** | **FFT Recognized** | | **FFT_%** | | **CFMT** | **PI-20** |  |  |
| --- | --- | --- | --- | --- | --- | --- | --- | --- |
| Exp1/1 | 34 | | 20.59 | | 53 | 67 |  |  |
| Exp1/2 | 32 | | 34.38 | | 56 | 69 |  |  |
| Exp1/3 | 34 | | 50 | | 42 | 38 |  |  |
| Exp1/4 | 26 | | 15.38 | | 36 | 42 |  |  |
| Exp2/1 | 35 | | 40 | | 42 | 85 |  |  |
| Exp2/2 | 48 | | 37.5 | | 34 | 73 |  |  |
| Exp2/3 | 49 | | 83.67 | | 41 | 65 |  |  |
| Exp2/4 | 54 | | 50 | | 50 | 68 |  |  |
| Exp2/5 | 24 | | 37.5 | | 34 | 56 |  |  |
| Exp2/6 | 20 | | 65 | | 36 | 72 |  |  |
|  | |  | |  | |  |  |  |
|  | |  | |  | |  |  |  |

Table 2. Single-case deficit analyses controlling for age (Exp. 3). Raw scores are shown for each potential DP case. P-values are one-tailed in the direction of expected impairment (lower scores for tasks; higher scores for PI-20), with age included as a covariate using the control sample (n = 16). Abn (%) indicates abnormality percentage (the estimated percentage of the control population expected to obtain a score as extreme or more extreme in the impairment direction). Asterisk denotes significance at p < .05 level.

| **Participant ID** | **CFMT** | | | **FFT** | | | **OFMT** | | | **GFMT2** | | | **PI20** | | |
| --- | --- | --- | --- | --- | --- | --- | --- | --- | --- | --- | --- | --- | --- | --- | --- |
|  | **Raw** | **p** | **Abn (%)** | **% correct** | **p** | **Abn (%)** | **Raw** | **p** | **Abn (%)** | **Raw** | **p** | **Abn (%)** | **Raw** | **p** | **Abn (%)** |
| Exp1/1 | 53 | .17 | 16.73 | 20.6 | <.01* | 0.02 | 139 | .02* | 2.41 | 65 | .03* | 3.15 | 67 | <.01* | 0.10 |
| Exp1/2 | 56 | .25 | 25.24 | 34.4 | <.01* | 0.13 | 136 | .01* | 1.41 | 69 | .15 | 14.85 | 69 | <.01* | 0.06 |
| Exp1/3 | 42 | .02* | 2.47 | 50.0 | .01* | 1.34 | 147 | .09 | 9.03 | 60 | <.01* | 0.33 | 58 | .01* | 0.72 |
| Exp1/4 | 36 | .01* | 0.72 | 15.4 | <.01* | <0.01 | 133 | .01* | 0.81 | 73 | .44 | 43.77 | 42 | .16 | 15.60 |
| Exp2/1 | 50 | .26 | 26.03 | 62.3 | .18 | 18.27 | 134 | .02* | 1.96 | 71 | .40 | 40.30 | 68 | .01* | 0.87 |
| Exp2/2 | 42 | .05 | 5.44 | 35.1 | <.01* | 0.30 | 130 | .01* | 0.71 | 61 | .01* | 0.78 | 85 | <.01* | <0.01 |
| Exp2/3 | 36 | .06 | 5.56 | 56.5 | .17 | 16.79 | 136 | .05* | 4.78 | 61 | .02* | 1.90 | 72 | .02* | 1.64 |
| Exp2/4 | 34 | .02* | 2.36 | 40.0 | .01* | 1.36 | 141 | .08 | 7.75 | 60 | .01* | 0.82 | 56 | .15 | 15.10 |

Table 3. Single-case dissociation analyses controlling for age (Exp. 3). Each suspected DP case was compared to the control sample. Dissociations between task pairs were then tested using the Revised Standardized Difference Test on age-adjusted values. T-values refer to the t-equivalent statistic for the discrepancy (df = 15), p-values are two-tailed. Abn (%) indicates the estimated percentage of the control population expected to show a discrepancy as large or larger in the observed direction; 'Worse' indicates the task that showed relatively poorer performance. Asterisk denotes significance at p < .05 level.

| **Participant ID** | **CFMT and FFT** | | | | **CFMT and OFMT** | | | | **FFT and OFMT** | | | | **OFMT and GFMT2** | | | |
| --- | --- | --- | --- | --- | --- | --- | --- | --- | --- | --- | --- | --- | --- | --- | --- | --- |
|  | **t** | **p** | **Abn (%)** | **Worse** | **t** | **p** | **Abn (%)** | **Worse** | **t** | **p** | **Abn (%)** | **Worse** | **t** | **p** | **Abn (%)** | **Worse** |
| Exp1/1 | 4.38 | <.01* | 0.03 | FFT | 1.12 | .28 | 14.1 | OFMT | 3.21 | .01* | 0.3 | FFT | 0.17 | .87 | 43.4 | OFMT |
| Exp1/2 | 3.55 | <.01* | 0.2 | FFT | 1.69 | .11 | 5.6 | OFMT | 1.55 | .14 | 7.1 | FFT | 1.61 | .13 | 6.6 | OFMT |
| Exp1/3 | 0.39 | .70 | 35.1 | FFT | 0.71 | .49 | 24.4 | CFMT | 1.36 | .19 | 9.7 | FFT | 2.09 | .05 | 2.7 | GFMT2 |
| Exp1/4 | 2.78 | .01* | 0.7 | FFT | 0.06 | .95 | 47.7 | CFMT | 3.01 | .01* | 0.4 | FFT | 3.02 | .01* | 0.4 | OFMT |
| Exp2/1 | 0.33 | .74 | 37.2 | FFT | 1.53 | .15 | 7.3 | OFMT | 1.69 | .11 | 5.6 | OFMT | 2.35 | .03* | 1.6 | OFMT |
| Exp2/2 | 1.79 | .09 | 4.7 | FFT | 1.02 | .32 | 16.2 | OFMT | 0.54 | .60 | 30 | FFT | 0.06 | .96 | 47.8 | OFMT |
| Exp2/3 | 0.89 | .39 | 19.3 | CFMT | 0.09 | .93 | 46.6 | OFMT | 1.06 | .31 | 15.4 | OFMT | 0.62 | .54 | 27 | GFMT2 |
| Exp2/4 | 0.36 | .73 | 36.3 | FFT | 0.65 | .53 | 26.3 | CFMT | 1.24 | .23 | 11.7 | FFT | 1.45 | .17 | 8.4 | GFMT2 |
